# Bending Setups for Reliability Investigation of Flexible Electronics

**DOI:** 10.3390/mi12010078

**Published:** 2021-01-13

**Authors:** Rafat Saleh, Maximilian Barth, Wolfgang Eberhardt, André Zimmermann

**Affiliations:** 1 Hahn-Schickard, Allmandring 9b, 70569 Stuttgart, Germany; Maximilian.Barth@Hahn-Schickard.de (M.B.); Wolfgang.Eberhardt@Hahn-Schickard.de (W.E.); zimmermann@ifm.uni-stuttgart.de (A.Z.); 2 Institute for Micro Integration (IFM), University of Stuttgart, Allmandring 9B, 70569 Stuttgart, Germany

**Keywords:** flexible electronics, mechanical characterization, bending, bending reliability, static bending, dynamic bending, bending apparatus, push to flex, roll to flex, three-point bending, four-point bending

## Abstract

Flexible electronics is a rapidly growing technology for a multitude of applications. Wearables and flexible displays are some application examples. Various technologies and processes are used to produce flexible electronics. An important aspect to be considered when developing these systems is their reliability, especially with regard to repeated bending. In this paper, the frequently used methods for investigating the bending reliability of flexible electronics are presented. This is done to provide an overview of the types of tests that can be performed to investigate the bending reliability. Furthermore, it is shown which devices are developed and optimized to gain more knowledge about the behavior of flexible systems under bending. Both static and dynamic bending test methods are presented.

## 1. Introduction

**Figure 1:**
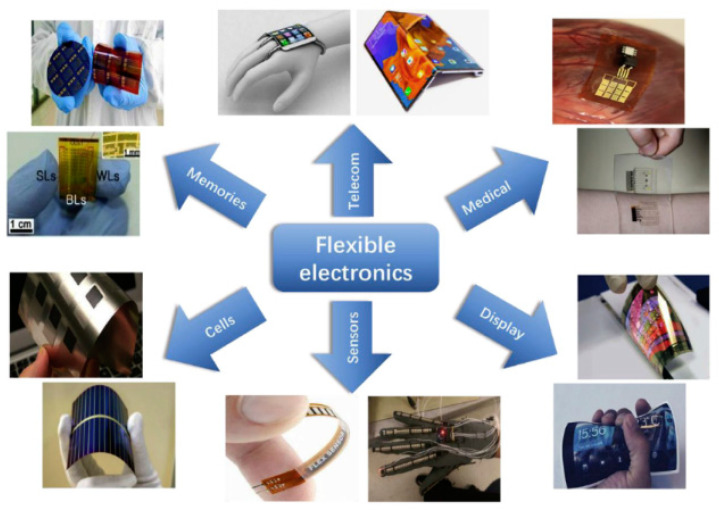
Flexible electronics and some of their applications in daily life [11].

The market for flexible electronics is growing steadily [1,2,3]. Reasons for the growing numbers of flexible electronics used include mechanical flexibility, high scalability, low weight, and, last but not least, large-area production compatibility [4,5,6]. Because of these advantages, flexible electronics find more applications and use in the industry in many sectors [6]. It is estimated that the global market for flexible electronics will be around 87.21 billion USD in 2024. In the Asia Pacific region alone, the market is expected to grow from about 5 billion USD in 2013 to 30 billion USD in 2024 [7]. Furthermore, according to a study by IDTechEx, the global demand for flexible hybrid electronics will reach a value of over $3 billion in 2030 [8]. Applications of flexible electronics include consumer electronics [9,10], industry technology [11,12,13,14], healthcare [15,16,17], automotive technology [4,18], and aerospace technology [18,19] (Figure 1). These sectors drive the growth of the market for flexible electronics. Related to flexible electronics, there is also a trend to foldable and stretchable electronic systems, which can be used in applications such as wearable and implantable electronics in healthcare, displays in consumer electronics, and robotic skin in industry applications [20].

Established semiconductor technologies can be integrated into flexible electronics, so that this combination has the ability to be bendable, deformed into irregular shapes, or even stretched [15,21]. Flexible electronics consist of electronic components, such as surface-mounted devices (SMDs) or ultra-thin chips on flexible substrates [22,23]. The electronic components are not flexible by nature but are as thin as possible to provide flexibility as a complete system when integrating onto or in flexible substrates [23,24]. Bending strain is one of the main movements inducing cracks and leading to the malfunction of flexible structures [20]. The mechanical reliability of these flexible electronics is very important to ensure the growing market demand for those systems. Furthermore, the robustness of the components and their integration should be optimized [25,26]. Ultra-thin chips are very common in flexible electronics and therefore need to be strong enough to withstand the tension that occurs during production and handling [12]. The integration of these ultra-thin and bendable integrated circuits in roll-to-roll processes enables the development of cheap, mechanically flexible electronics. To make these systems reliable, detailed investigations of both the strength and the deformation of the layers of thin components have to be performed. Once these properties are known, individual technology steps can be characterized and optimized in the best possible way [27].

Flexible electronics must be able to deform and at the same time, both the functional properties and the electronic parameters must remain unaffected by bending during application [19]. Moreover, with growing demands on the reliability of flexible systems, the need for characterization of the mechanical stability of the components also increases. [28]. Therefore, the testing of their reliability under mechanical bending is essential [29,30]. The goals of the bending test are to understand the behavior of flexible systems under bending and their fatigue stability.

The failures associated with bending strongly influence the reliability of the components. This failure is mainly caused by the forces generated due to bending or vibration of the circuit carriers [31].

This paper reviews the mechanics and methods of reliability testing for flexible electronics in bending tests in order to provide an overview of existing research in the field.

## 2. Substrates for Flexible Electronics

Flexible systems require mechanically compliant substrates that adapt to soft and irregularly shaped surfaces. Furthermore, large area compatibility, chemical stability, process-compatible melting and glass transition temperatures, outgassing rates as well as low surface roughness are very desirable properties for flexible systems [19,32]. Table 1 gives an overview of the most used substrates in flexible electronics and some useful properties.

## 3. Application of Flexible Electronics

### 3.1. Wearables and E-Textiles

**Figure 2:**
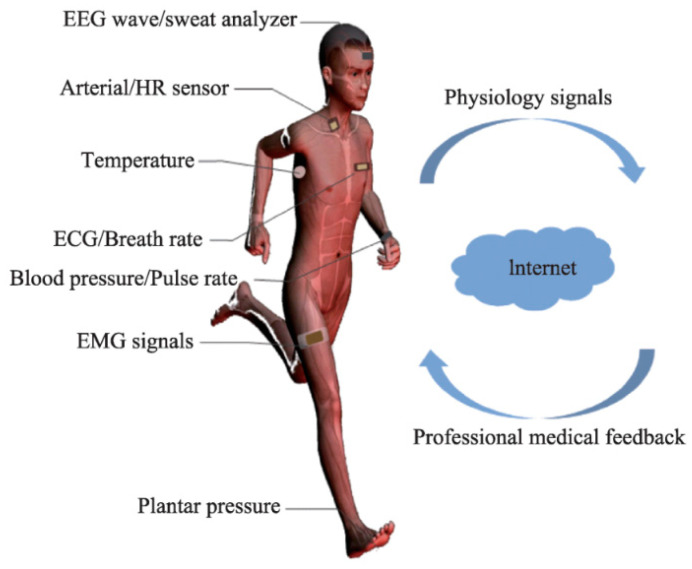
Monitoring of some physiological signals using flexible sensors and diagnosis and evaluation per remote medicine. Adapted from [41]. The abbreviations EEG, HR, ECG, and EMG stand for electroencephalogram, heart rate, electrocardiogram, and electromyogram, respectively.

Flexible electronics are increasingly used in wearables and smart medical applications. Medical resources are reaching their limits when it comes to providing for our ageing society. Traditional medical methods cannot meet the needs of patients in time. Flexible and portable health monitoring, on the other hand, offers a completely new technology and alternative to traditional diagnostic methods. At the same time, health care will be portable and timely [41]. Figure 2 illustrates some physiological signals, which can be measured using wearable flexible sensors and then remotely evaluated.

Someya et al. [9] developed an electronic artificial skin based on large-area flexible pressure sensors with field-effect transistors built on a flexible substrate. Lumelsky et al. [42] worked on a large-area, flexible array of sensors, consisting of LEDs and detectors, which can cover the entire surface of the human body or a machine and has the ability to detect many signals such as pressure, temperature, or even touch. Similar to Lumelsky, Manssfeld et al. [43] published a paper on an electronic system using large arrays of capacitive pressure sensors with excellent sensitivity and very short response times on a flexible and stretchable polydimethylsiloxane (PDMS) substrate.

Compared to polymer foils, textile structures have additional potential for being stretched and deformed, while also allowing for a degree of breathability. For this reason, they can adapt well to the shape of the body. By integrating flexible sensors in textiles, namely, e-textiles, numerous applications can be realized, especially in the healthcare sector [32]. A market study by IDTechEx has predicted that the market for e-textiles will be worth over $1.4 billion by 2030 [44].

**Figure 3:**
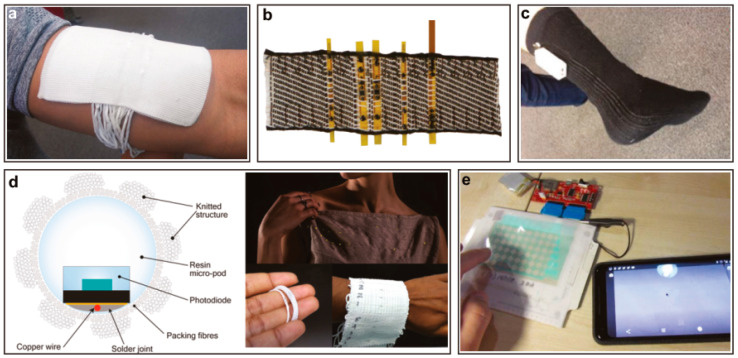
Examples for smart textiles and their applications [32]. (**a**) Temperature sensing yarns in a textile for health monitoring [45]. (**b**) Optoelectronic near-infrared spectroscopy smart textile for measuring the blood oxygenation levels in health care [46]. (**c**) A temperature-sensing sock for fitness and health care [47]. (**d**) Embedded photodiodes in textile for health-monitoring [48]. (**e**) Tactile-sensing fabric for applications in human-machine interfaces (HMIs), smartphones, and Internet of Things (IoT) devices [49].

Figure 3 shows some examples of integrated flexible sensors in textiles and yarns. With the help of these sensors, vital parameters of the human body can be measured and further evaluated remotely.

An et al. [50] reported on the fabrication and characterization of a transparent and flexible fingerprint sensor array with multiplexed detection of tactile pressure and skin temperature for mobile devices and smartwatches. The sensor is built based on a silver nanofiber and fine silver nanowires (AgNWs) on a flexible polyimide substrate [50].

### 3.2. Flexible Displays

**Figure 4:**
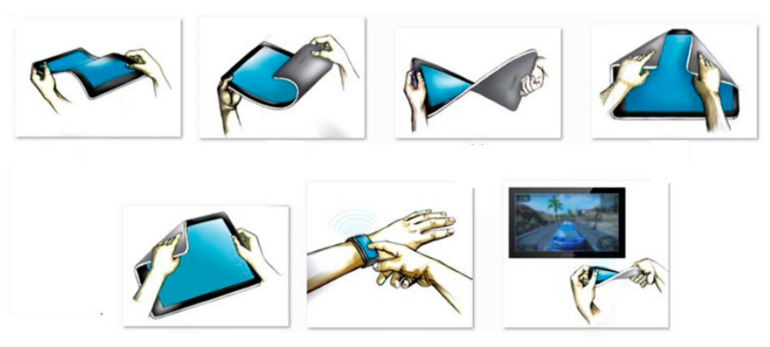
Handling of flexible devices and displays. Adapted from [54].

Optimization of organic light-emitting diodes (OLEDs) leads towards mechanical flexible displays when integrated on plastics. This is possible due to the unique properties of OLEDs, including their ultra-thin and simple structure and low-temperature manufacturing process [51]. Forrest [52] reviewed the organic semiconductors used to fabricate flexible displays. Moreover, he covered the technologies for the deposition of polymer thin films. To reduce costs in the production of large-area flexible displays, Gelinck et al. [53] demonstrated the use of organic transistors on flexible substrates for the development of flexible active-matrix monochrome electrophoretic displays. Figure 4 demonstrates the possible handling of flexible displays.

Im et al. [55] reported on a process chain for fabricating a mechanically flexible OLED on flexible plastic substrate. A high performance conductive film with an embedded transparent conductive electrode of copper nanowires was used [55]. An attractive application for flexible displays is their integration into clothing and textiles. Ivanov et al. [56] have investigated the use of light-emitting diodes (LEDs) and printed electroluminescent elements to fabricate flexible displays for integration into textiles. Huang et al. [57] also reported on the integration of flexible displays into textiles.

### 3.3. Diagnostics and Healthcare

**Figure 5:**
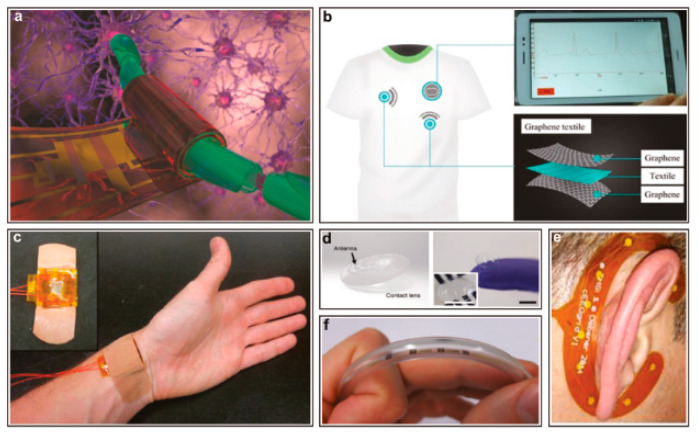
Applications for bio-monitoring, diagnosis, and hazards prevention [9]. (**a**) Flexible electronics for regenerative neuronal cuff implants [62]. (**b**) Flexible graphene wearable electrodes for dynamic ECG sensing [63]. (**c**) Flexible polymer transistors for application in electronic skin and health monitoring [64]. (**d**) Contact lens with ocular diagnostics of detecting the glucose levels in tears in diabetes patients [65]. (**e**) Ambulatory and wearable EEG sensor around the ear [66]. (**f**) Electronic nose on a flexible substrate for detecting hazardous gases [67].

Flexible electronics gain more interest in medical sectors because they offer more comfort, while being in contact with patients [58]. In the early 2000s, Nathan et al. [59] already reported on the use of large-area flexible electronics for large area X-ray imaging. They produced thin-film electronics on flexible polymer substrates. Both Jin et al. [60] and Ko et al. [61] published papers on the fabrication of electronic eye cameras on flexible polymer substrates. Figure 5 shows some applications for flexible electronics in the healthcare sector.

An interesting approach for health monitoring and, for example, the realization of an e-skin, is the fabrication of active-matrix pressure sensors. These consist of integrated arrays of graphene transistors and can measure pressure up to higher-pressure ranges up to 3 MPa [68]. Other new research in medicine and electronics is the use of virtual reality (VR). Li et al. [41] provided an overview of the use of flexible sensors for health monitoring in virtual reality. This trend in the use of flexible sensors is revolutionizing medicine and especially telemedicine. Flexible sensors can be applied to the skin, enabling personalized medicine by collecting important parameter data from the human body, and capturing meaningful changes in health status [69]. Gao et al. [69], Khan et al. [70], and Wang et al. [71] reviewed the current researches and applications of flexible sensors to measure the vital signs of the human body.

## 4. Mechanics of Bending

**Figure 6:**
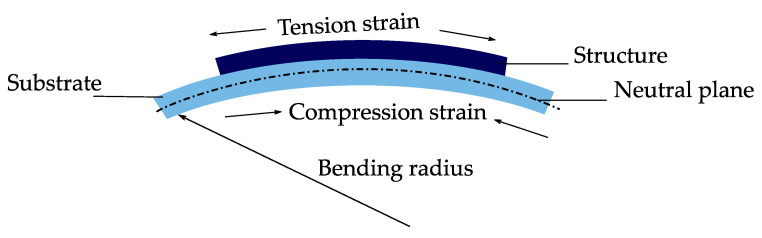
When bending a structure built on a substrate with a given radius, the outer plane will be under tension and the inner plane under compression strain.

When flexible components are integrated on or into flexible or even stretchable substrates, new mechanical tests of the system are essential. Consequently, the mechanical reliability of flexible electronics becomes one of the critical aspects of this technology [72]. When bending a flexible system, which consists of a thin structure on a substrate with a radius *R*, the top area of the structure will be under tension, while the other side is under compression (Figure 6) [73]. In this case, the upper surface undergoes elastic tension strain, and the lower surface elastic compression strain. The neutral plane, which describes the surface inside the film, does not undergo any elongation [20,73]. When designing the flexible system the strain within the system can be improved by placing the rigid components away from the surface of the substrate, where the bending strain is largest, to the point of the neutral plane where the strain is minimal [15]. At this point, it is worth mentioning that the neutral plane in the film/substrate composite is not equal to the neutral plane in a homogeneous material if there is any mechanical mismatch between the components [74]. This point has to be considered when designing flexible electronics. Palavesam et al. [25,29] compared the breaking strength of bare thin chips and chips embedded in flexible foil. They found that chips embedded in flexible film substrates have a higher breaking strength, up to 80% more than bare chips. This means that very small bending radii are possible due to the embedding. Kim et al. [10] also found similar results when they examined the reliability of systems on flexible substrates under a three-point bending test.

When bending a substrate with thickness *t* on a defined radius *R*, the surface strain ε can be calculated based on the bending theory of thin films as below [58]:

(1) $$\varepsilon =\frac{0.5\cdot t}{R+0.5\cdot t}$$

Since the ultra-thin chips are mechanically very flexible and can achieve a deflection higher than their thickness, resulting in very small bending radii, their bending stress cannot be evaluated with classical beam theory. However, the investigation of this bending stress and achievable bending radii is of great relevance, since this information is the key to the design and manufacture of more robust and reliable flexible electronic systems [25,29].

The mechanical properties, such as breaking strength, breaking displacement, and breaking stress, can be investigated by applying mechanical tensile stress to the flexible electronics. The breaking strength describes the load on the component at which the component is subjected to the mechanical stress breaks. The reason for this stress can be mechanical as well as thermal load. As a result, a displacement occurs at this breaking strength, which is called fracture displacement [25,29]. However, applying a pure tensile stress is complicated because the components integrated in flexible electronics have very small dimensions. Nevertheless, in order to be able to investigate the mechanical properties of the components, uniaxial bending tests can be performed to apply a line load to the components. There are two different types: the three-point bending test and the four-point bending test [25].

The full understanding of the mechanical stability of the individual components of a flexible system enables the development of reliable applications. This requires the investigation of the minimum bending radius and the load limit of the components before breakage. These can be determined primarily with the three-point bending method [75].

### 4.1. Three-Point Bending

**Figure 7:**
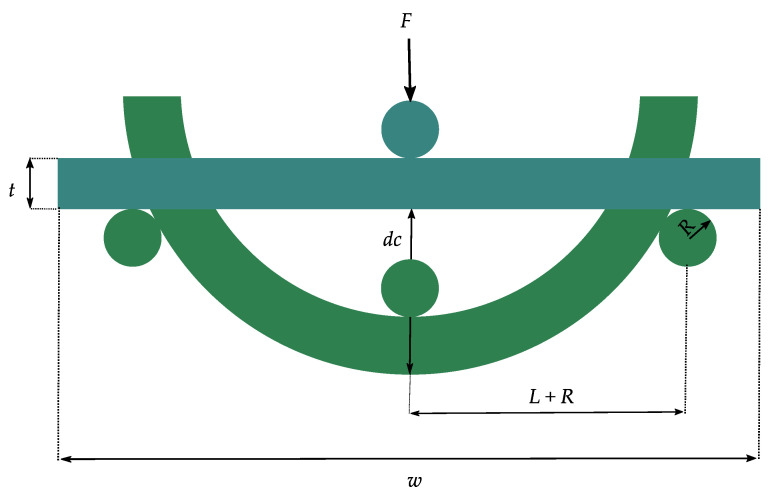
Schematic view of three-point bending setup.

Three-point bending measurement is usually used for rigid and semi-flexible components to evaluate their maximal mechanical fracture stress and stability under bending [30,76]. When testing flexible ultra-thin chips many factors should be considered to understand the behavior of the load-displacement function [28,30]. During the mechanical investigation of the thin chips under three-point bending, many factors strongly influence the measurement results. These factors include the edge radius of the supports used to hold the chips in place and asymmetries in the structure, namely, the variation of the distance between the outer support and force application position (Figure 7) [30].

One major advantage of the three-point bending measurement is the possibility to obtain the stress-strain relationship and with this information, the behavior of the flexible electronic response to an applied load can be predicted [77,78]. Moreover, this bending measurement can help to determine Young’s modulus of the component [78].

While bending, the occurring mechanical stress σ generally depends on the bending moment M and the moment of inertia of the system I to be bent, but also on the distance from the neutral plane *z* [28]. The following Equation (2) describes the relationship. With increasing distance from the neutral plane the occurring mechanical stress increases until it reaches its maximum at the largest distance of *z* [28,79]:

(2) $$\sigma =M\cdot \frac{z}{I}$$

Different from the previous Equation (2), the mechanical stress for a sample whose deformations during bending are smaller than its thickness can be calculated as follows [80,81]. For such a sample with length *l*, breaking stress *F*, width *w*, and thickness *t*, the Equation (3) describes the stress that occurs:

(3) $$\sigma =\frac{3}{2}\cdot \frac{F\cdot l}{\left(w\cdot t^{2}\right)}$$

The edge damage of a chip or a system is also considered in three-point bending. However, this measuring method overestimates the chip strength because the uniaxial force is only induced as a line in the middle of the chip and thus the exact break position is not exactly defined. For the exact calculation of the breaking strength, the exact break position is very necessary. This accuracy is especially important because the functional structures are unevenly distributed on the chip. Furthermore, the distances between the supports strongly influence the bending strength and lead to non-linear effects in the case of large curvature [13,26,27].

### 4.2. Four-Point Bendings

Four-point bending is also a common method for evaluating the adhesive strength of multi-material stacks, known as multi-flex layers [82].

**Figure 8:**
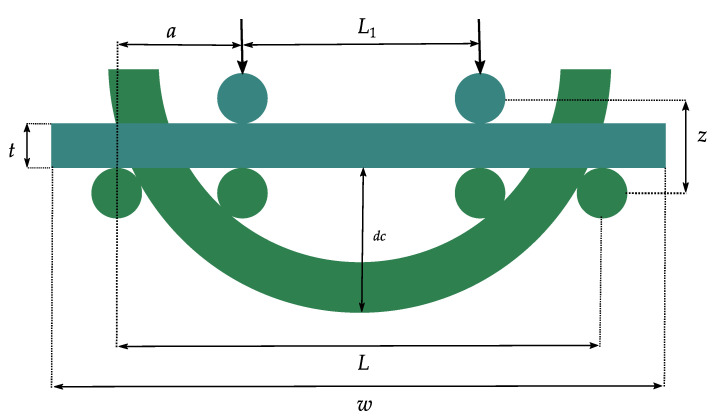
Diagram of a four-point bending setup before and after the bending.

In four-point bending, the upper supports are pressed on the material with a certain force *F* and thus move it by a deflection *z* for bending. The lower supports, on the other hand, are fixed (Figure 8). The thickness *t* and width *w* as well as material parameters, such as the modulus of elasticity *E*, determine the height of the deformation [13]. If thick components are bent to failure by means of a four-point bending, so that the achieved bending deflection *z* is very small compared to distance *a* between the inner and outer supports, the maximum bending stress *σ* occurring in the center of the component can be calculated by Equation (3) using the usual bending formula for smaller deflection as follows [13,83]:

(4) $$\sigma =\frac{3\cdot F\cdot a}{\;w\cdot t^{2}}$$

If the bending deflection *z* and the modulus of elasticity *E* of the component are known, the current bending stress between the inner supports can be calculated by Equation (5) [13,83]:

(5) $$\sigma =\frac{-3\cdot z\cdot E\cdot w}{a\cdot \left(3\cdot l-4\cdot a\right)}$$

The increasing demand for miniaturization of integrated circuits led to the comprehensive investigation of three-dimensional (3D) integration. The mechanical stress induced by this, for example, by stacking and wafer thinning, has a significant impact on the properties of the components involved [84].

The previous equations are mainly used when thick components with a thickness above 100 µm and very little bending deflection (z << *a*, Figure 8) are tested under four-point measurement. If, on the other hand, thin components are tested with bending deflection greater than their thickness, the curvature *k* can be calculated in Cartesian coordinates by Equation (6), assuming a linear elasticity. Here the curvature is a function depending on the position *x* [13,85]:

(6) $$k=\frac{1}{r}=\frac{d^{2}y/d_{x^{2}}}{\;{\left(1+{\left(d_{y}/d_{x}\right)}^{2}\right)}^{3/2}}\;$$

where *r* is the local bending radius. If the linear bending theory is assumed and the ratio *d_y_/d_x_* is close to 1, then the maximum strain ε is calculated by Equation (7) [13,86]:

(7) $$\varepsilon =\frac{y}{r}$$

where *y* describes the distance from the center of the chip. Based on Equations (6) and (7), the mechanical stress σ at high curvature can be derived from the radius of curvature *r* of the component (Equation (8)):

(8) $$\sigma =E\cdot \frac{y}{r}$$

### 4.3. Push and Roll to Flex

The widely established and standardized test procedures, such as the three-point bending test, are more suitable only for small displacements and especially large bending radii of the systems [72]. For this reason, other test methods are suitable, which allow higher displacements with very small bending radii. These methods include characterization test procedures such as the push or roll to flex bending test.

The roll to flex bending measurement allows, by rolling the materials over different diameters of a cylinder, to study the mechanical properties of the materials at different cycled strains. The thickness or diameter of the materials and the diameter of the roll control these induced strains [87].

**Figure 9:**
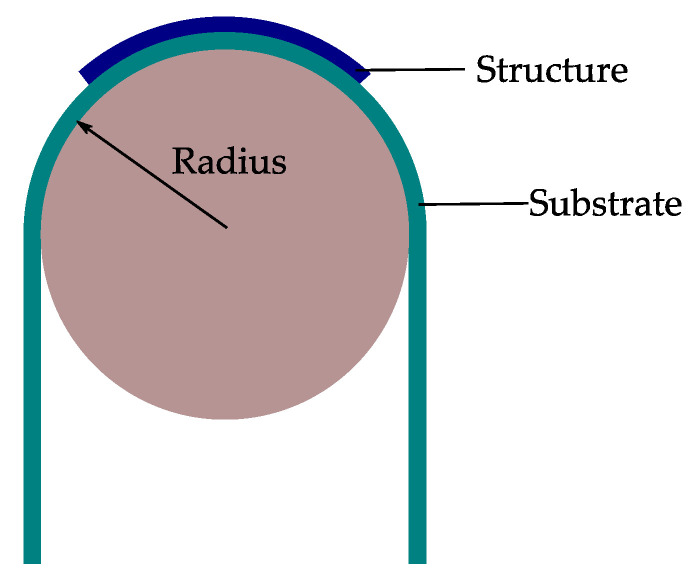
Principle sketch shows a system bent over a defined bending radius. With Equation (10), the bending stress $\sigma_{str}$ at the top of the structure can be calculated.

When flexible components like ultra-thin chips or structures with a thickness $t_{str}$ bend over a supporting substrate $t_{sub}$ over a defined radius *R*, the strain $\varepsilon_{top}$ on the top surface of flexible structures can be calculated by Equation (9) [20,73,88] (Figure 9):

(9) $$\varepsilon_{top}=\frac{\left(t_{str}+t_{sub}\right)}{2\cdot R}\;\frac{\left(1+2\eta +\chi \eta^{2}\right)}{\left(1+\eta \right)\left(1+\chi \eta \right)}$$

where η=tstrtsub, χ=YstrYsub and Y the Young’s modulus of the material.

(10) $$\sigma_{str}=E_{str}\cdot \varepsilon_{top}$$

where $E_{str}$ describes the elasticity modulus of the structure and $\varepsilon_{top}$ the strain on the top. Putting Equation (9) in Equation (10) results in Equation (11):

(11) $$\sigma_{str}=E_{str}\cdot \frac{\left(t_{str}+t_{sub}\right)}{2\cdot R}\cdot \frac{\left(1+2\cdot \frac{t_{str}}{t_{sub}}+\frac{E_{str}}{E_{sub}}\cdot \frac{t_{str}{}^{2}}{\;t_{sub}{}^{2}}\right)}{\left(1+\frac{t_{str}}{t_{sub}}\right)\left(1+\frac{E_{str}}{E_{sub}}\cdot \frac{t_{str}}{t_{sub}}\right)}$$

After solving Equation (11), the formula for calculating the bending stress can be simplified (Equation (12)):

(12) $$\sigma_{str}=E_{str}\cdot \frac{1}{2\cdot R}\cdot \frac{E_{str}\cdot t_{str}{}^{2}+E_{sub}\cdot t_{sub}\cdot \left(2\cdot t_{str}+t_{sub}\right)}{E_{str}\cdot t_{str}+E_{sub}\cdot t_{sub}}$$

According to Equation (9), if the substrate and material thicknesses are well chosen, the strain of the structures can be controlled for known bending radii. With the selection of suitable materials and from Equation (9) it can be seen that mainly the layer thicknesses and the bending radius rather dominate the strain [89]. However, if no strain is desired at all, then the structures should be encapsulated between two substrates, for example, the structures lie in the neutral plane. The latter is secured if the ratio of material thickness and Young’s modulus of both substrates is the same [20,73]. At this point, it is worth mentioning that the mechanics of the structure of the film on the substrate depends strongly on Young’s modulus and thickness of the substrate and the film [90].

If the components attached to a foil are very thin and bent under the same bending radius, Equation (12) for calculating the strain can be simplified. In this case, the strain $\varepsilon_{top}$ caused by bending can be approximately calculated by Equation (13) [59,73,91,92,93,94].

(13) $$\varepsilon_{top}=\frac{\left(t_{str}+t_{sub}\right)}{2\cdot R}$$

Table 2 illustrates the strain occurring for a certain exemplary combination of material thicknesses and bending radii based on Equation (13).

## 5. Bending Machines

This section provides an overview of different bending machines for bending measurement of flexible electronics.

Depending on the type of bending during application, be it once or repeated bending or even stretching, the requirements for investigation of the systems are different. For this reason, understanding of the mechanical and electrical properties of the structures under externally applied strain is necessary [90].

### 5.1. Static versus Dynamic Bending

A static bending test is the simplest and mostly reported test to investigate the reliability of flexible electronics [6]. However, some applications in their function will not just be bent one time but also undergo dynamic bending. For those systems, static bending is not enough to get an understanding of the reliability of the product in later working conditions [94]. Therefore, consideration of dynamic bending is necessary.

A static bending test can be performed to understand the behavior of the system under internal stress. According to the application, the static bending test is to be performed in concave or convex orientation. In the concave orientation, the components on a substrate are subjected to tensile stress, while in the convex orientation they are subjected to compressive stress [10].

**Figure 10:**
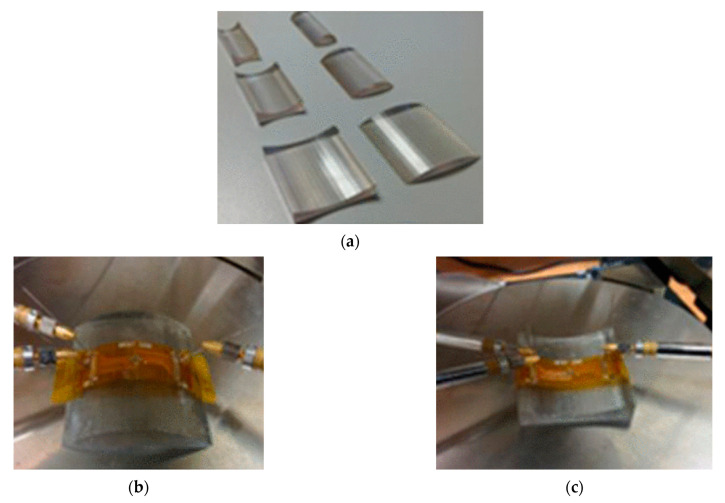
(**a**) 3D printed structures for static bending test, (**b**) convex (tensile) and (**c**) concave (compressive) bending setup of thin chip on flexible foil. Adapted from [92].

For the procedure of a static bending test, the flexible system will be bent over a rod or a tube with a defined radius (Figure 9). Dependent on the kind of mechanical stress of the system, two categories are possible. Concave bending, where the system is bent on the inner area of the tube, in this way the system will be compressed and get compressive stress [95]. On the other hand, in convex bending, the system is bent on the outer area of a rod. Compared to concave bending, the system gets tensile stress (Figure 10) [6,90,96].

Based on type of structures and materials applied on substrates some structures are more sensitive to tension than to compression. The bending test should be designed and chosen with regard to the intended application. Both active and passive flexible electronics can go through static and dynamic bending tests to investigate their stability under bending. Moreover, an understanding of the critical bend ratio can be gained in this manner. Bend ratio describes the bending ratio to substrate thickness [6].

In applications where the flexible electronics will undergo very few bending cycles, the investigation of static bending reliability can be sufficient. In contrast, there are applications where flexible electronics are used to make the electrical connection to movable parts, such as the display of mobile flip phones or computers. Even the connection to fast moving parts with very high lifetime cycles, for example, read heads of optical drives, is achieved using flexible film connections. The investigation of the dynamic bending reliability of the components is indispensable [97].

### 5.2. Three-Point Bending versus Four-Point Bending

Three-point bending measurement is frequently used to investigate the stability and flexibility of ultra-thin chips [12,98]. A ball-and-ring test can be used to examine stability. A force-giving system presses the ball onto the chip and moves slowly, while a computer records data, such as force and distance until the chip breaks. In another variation to investigate the flexibility, the chip is placed on two rods. The force-giving system presses the third rod on the chip until the chip cracks [12].

**Figure 11:**
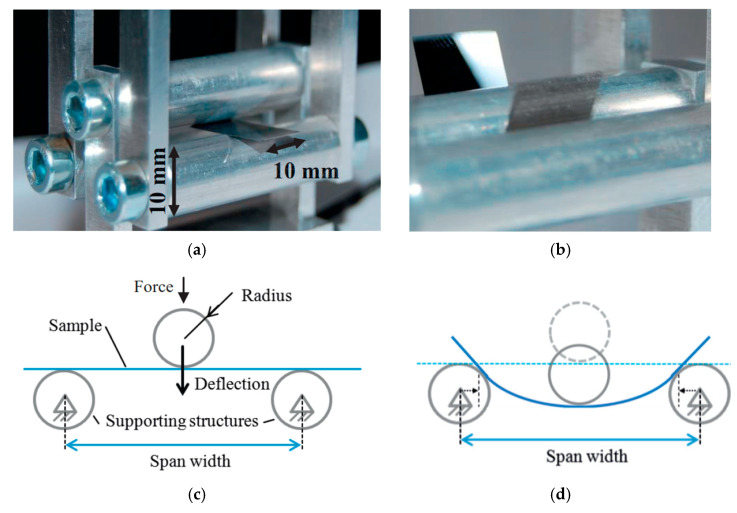
Experimental setup in three-point bending. (**a**) Perspective view; (**b**) when bending with large deflection; (**c**) diagram of the sample in plain position and (**d**) with a deformed position of the sample. Adapted from [78].

Both three-point and four-point bending measurements are based on measuring the chip curvature during bending and calculating the resulting bending stress from the measured curvature [13]. Figure 11 shows a three-point bending setup.

### 5.3. Push to Flex versus Roll to Flex Bending

Both push and roll to flex bending setups are more commonly used for foil based systems. In this case, the change in properties of the functional structures on the substrate is used as measure for damage to these structures. For example, the change in resistance of the structure is monitored as a function of the bending cycles. If the resistance rises above a fixed percentage value compared to the initial resistance during the bending test, this is considered as a failure [14,94,99].

**Figure 12:**
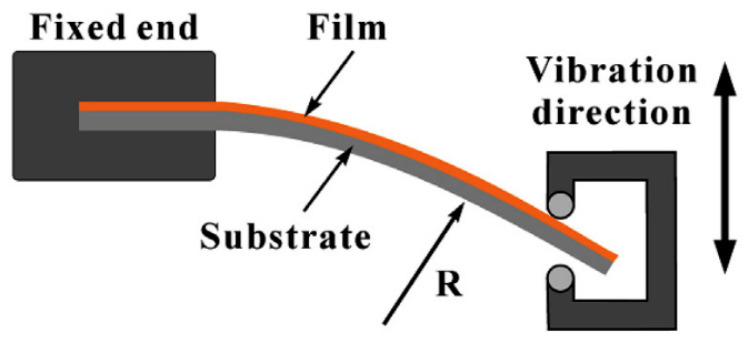
Dynamic bending setup with full reverse loading. Adapted from [74,100].

Figure 12 shows a setup for bending a cantilever and the like with a reverse load. It is shown that the deflection with the curvature *R* is a function of the horizontal distance. During deflection of the sample on such a bending setup, the strain is not distributed uniformly over the sample, but is at its maximum near the fixed end and decreases sharply along the length of the sample. This has shown that the local fatigue life along with the sample also varies due to strain gradients [100].

**Figure 13:**
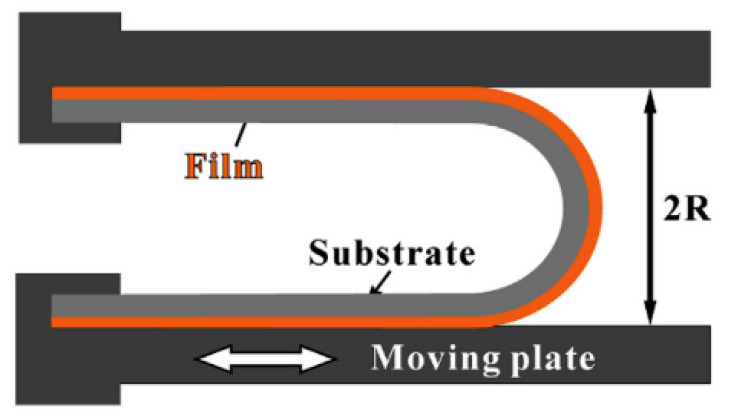
Schematic illustration of cyclic bending test system. Adapted from [37,74,91,96].

Figure 13 shows another setup to investigate the bending reliability of a sample. Two plates fix a sample at both ends and bend it in the middle with a gap of 2 × *R*. The upper plate is fixed, while the lower one is subjected to repeated linear movement. This induces fatigue damage in the sample. The strain in the curved area 2*R* is not uniform but has a gradient. However, the bending theories discussed in Section 4 can be used to approximate the strain, assuming uniform strain with constant radius [91].

**Figure 14:**
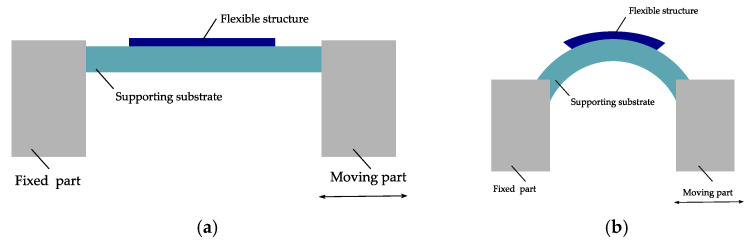
Schematic view of dynamic push to flex bending setup: (**a**) In a flat position and (**b**) under bending. Modified and freely sketched after [95,101].

Due to the low thickness of flexible electronics, it is necessary to use supporting substrates for using push to flex bending [10]. Otherwise, the radius will be undefined. This is one of the drawbacks of using this system to investigate the bending reliability. Moreover, due to the supporting system, the active system attached becomes more rigid than usual. Figure 14 shows a push to flex bending machine. In this bending machine, the flexible electronics are placed on a thicker supporting substrate.

The thicker substrate dominates the mechanical properties of the flexible system. Moreover, the bending radius is not strictly defined. The strain of the substrate has its maximum values in the area near clamped substrate ends [100].

**Figure 15:**
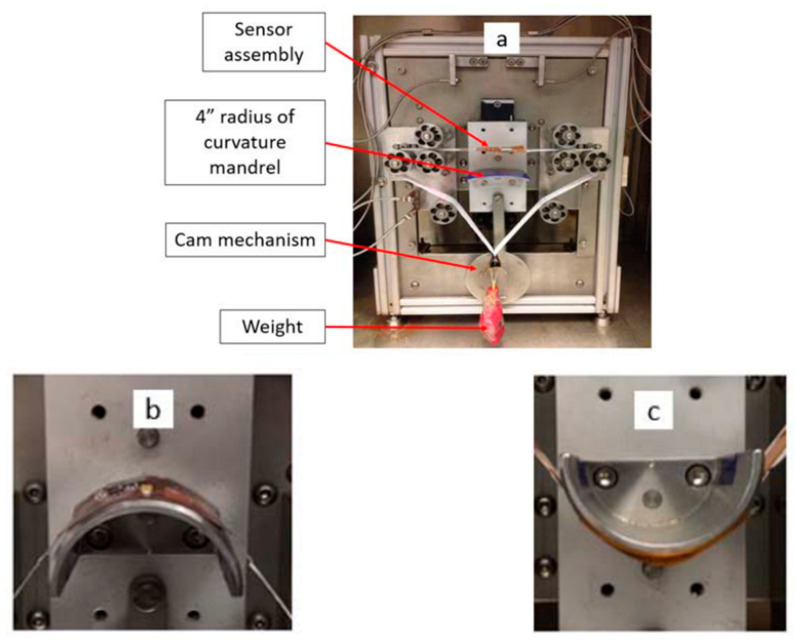
(**a**) Dynamic push to flex bend-testing setup; (**b**) components on the top are in tensile and (**c**) in compression (reproduced with permission) [102].

Soman et al. [102] report on an environmental flexure tester, Associated Environmental Systems (Model No. BHK 4108), to investigate their samples using a flexure test (Figure 15). The samples contain a component side and a sensor side. The flexure test is performed in two variations: in one, the bending mandrel presses against the sensor side so that the component side is under strain and vice versa in the other. The bending mandrel can run a number of bending cycles.

Hamasha et al. [22] report on a test setup similar to Figure 15 to investigate the reliability of structures deposited by physical vapor deposition (PVD). The test setup consists of one or two bending mandrels. In the case of the variant with one mandrel, the sample is only pressed from one side during the upward stroke, so that one side is under tension and the other under compression. With the other variant, on the other hand, the sample is pressed from below and from above, so that alternating tensile/compressive forces are applied to the same side of the sample.

**Figure 16:**
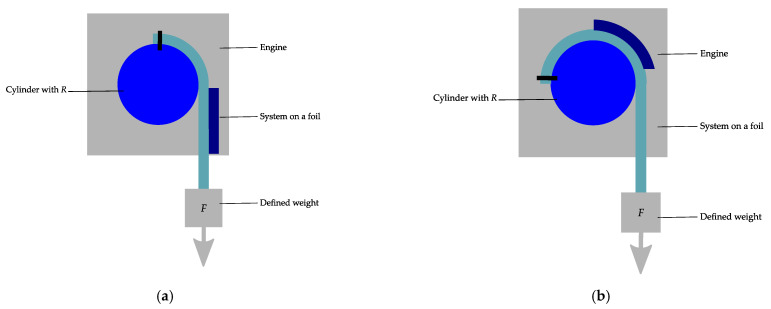
Illustration of a roll to flex bending machine with attached sample; (**a**) in a flat and (**b**) in a bent position. Modified and freely sketched after [94].

Wright et al. [94] report on a bending machine, which is used to examine the bending reliability of flexible electronics. The reliability investigation occurs under constant bending radius and defined tension. Figure 16 illustrates the concept of the machine. The substrate is fixed onto the rod. The substrates align with the radius of the rotating rod, while constant tension is applied by a weight.

**Figure 17:**
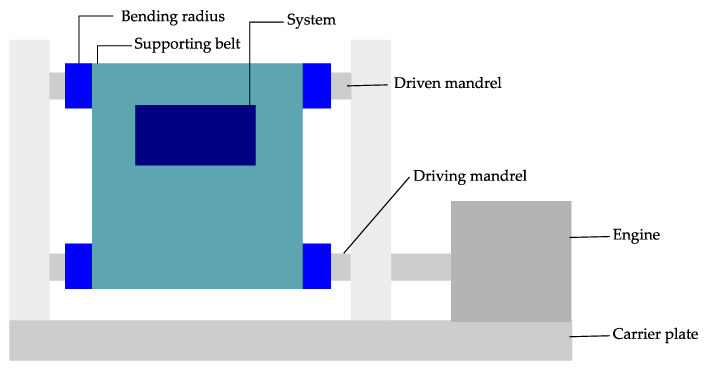
A cyclic bending tester with roll to flex method. Modified and freely sketched after [103].

Based on the roll to flex bending test, the bending machine shown in Figure 17 is also of interest.

A supporting belt with attached flexible electronics rotates completely over a roller. For applications where online measurements with cable connections are required, this setup is not ideal. Using this bending machine, Jeong et al. [103] investigated the bending reliability of the flexible near-field communication tag. This setup is perfect for this application, because the near-field communication tag does not need a cable while testing.

**Figure 18:**
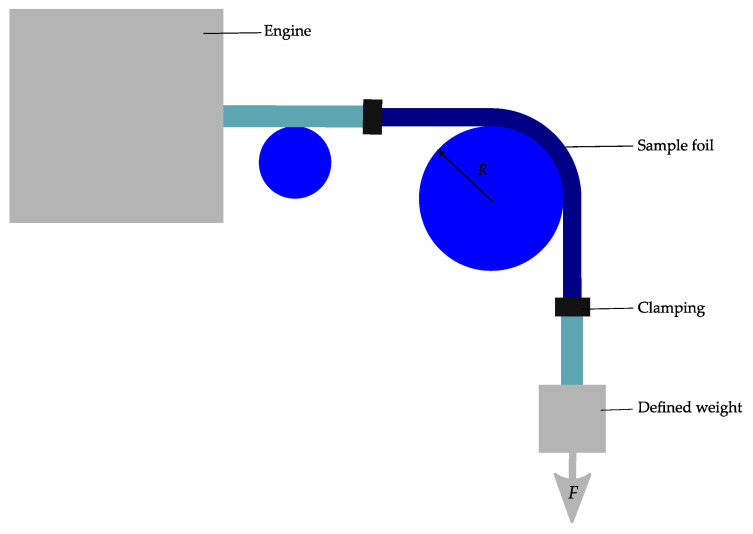
Dynamic bending machine. Modified and freely sketched after [93].

Another setup for bending reliability is built so that the foil or system to be bent is linked to strings at both ends. These strings are connected to a motor and a weight respectively. The foil with the rope is moved back and forth over a roller and bends (Figure 18) [93].

## 6. Discussion

### 6.1. Three- and Four-Point Bending

In the three-point bending measurement, the maximum stress occurs in the center of the sample to be tested. On the one hand, this can lead to unwanted failure. On the other hand, the functional structures may be distributed non-uniformly over the sample and thus experience irregular stress [13]. Compared to three-point bending, four-point bending has many advantages. It ensures a uniform bending stress between the two supports, which is desirable in many cases. In addition, the errors caused by misalignment remain small, so that good results are achieved [26,83]. It should be noted that the two methods apply to small displacements (few micrometers) and large radii of curvature [72]. For this reason, these methods are more suitable for bend testing of both individual components and thick systems.

### 6.2. Push to Flex Bending

The stiffness of the carrier substrate as well as the mounted components on the foil influence the resulting bending radius during the bending test of flexible electronics when testing with this method [94].

In test setups similar to those of Soman et al. [102] and Hamasha et al. [22], both static and dynamic bending tests of flexible electronics can be performed.

One of the disadvantages of this bending measurement method is the undefined bending radius, called freeform bending. The sample to be tested does not experience a constant bending radius. In addition, each position on the sample has a different bending radius. Furthermore, very small bending radii are difficult to achieve [6,104].

The advantages of the push to flex measuring method include the possibility of concave and convex bending tests with the same measuring device, since the sample is bent into the gap between the two ends and has no other contact with components. Furthermore, the bending test setup can be upgraded to a torsion test setup by adding a rotation axis to the moving ends. The push to flex bending setups are more suitable for applications with a thickness of over about 100 µm [95,99].

### 6.3. Roll to Flex Bending

During this measuring method, the flexible electronics and thus the mounted components on polymer foils are repeatedly bent over a cylinder and the bending radius remains constant during the entire measurement [94].

With the roll to flex bending measurement method, in contrast to the push to flex bending measurement method, the sample is bent onto a cylinder or roller, which means that constant and even small bending radii can be achieved. However, the sample must be loaded with some extra load to force the sample onto the curvature of the roller [6]. Although this load is necessary to provide tension to the system on the bend radius, it causes a tensile force on the film and the electronics mounted on it. This tensile force must be considered when calculating and evaluating the failure mechanisms of the tested electronics. Furthermore, concave bending of substrates without a protective layer is challenging with this bending method because the sliding on the roll surface can damage the thin functional structures [6].

To increase the quality of the bending investigation significantly, using a roll to flex bending, three factors should be observed and taken into account. These factors include the constant bending radius, the constant speed of the roll, and a defined tensile force [104]. The roll to flex bending setup is more suitable for thinner systems below 100 µm and is widely used in the field of foil based systems or system-in-foil [14,94].

**Figure 19:**
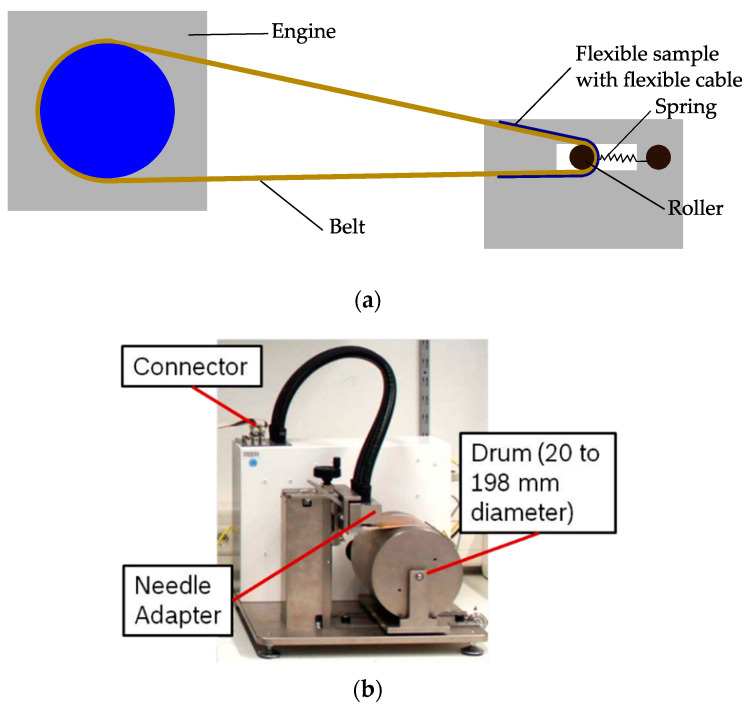
Examples of two bending test setups using the roll to flex bending measurement method with decoupled influence of tensile forces on the bent samples; (**a**) on-belt flex bending setups (modified and freely sketched after [6]) and (**b**) bending test device (reproduced with permission) [14].

Ideally, the bending test of flexible electronics is performed without any influence of built-in tensile forces and on constant bending radius. This can be guaranteed by bending the sample onto a roll, while the whole setup with the sample, in case of tensile forces, slackens so that this tensile force is compensated. Figure 19 shows two test rigs, which allow an investigation of the bending reliability without the influence of tensile forces. Figure 19a shows an on-belt setup. Here the sample is fixed on a flexible belt. The belt rolls around a bending roller, which slides over a spring construction and rail slot. The belt and springs absorb the resulting tensile forces [6]. Figure 19b shows another bending test setup. The sample is rolled onto a drum. The drum rotates and slides on the base plate so that the sample does not receive any tension [14,105].

## 7. Conclusions

In this paper, bending apparatus for flexible electronics are reviewed, and the basic theory and mechanics of bendable electronics are explained. Moreover, usually used substrates in flexible electronics and applications are briefly mentioned to provide a better understanding about flexible electronics and corresponding bending reliability investigation. Various frequently used methods such as dynamic and static, and push or roll to flex are discussed. Examples for bending machines for the different methods and their advantages and disadvantages are presented.

## Figures and Tables

**Table 1:** Overview of the most used plastic substrates for flexible electronics and some of their properties.

Material	Polyethylene Naphthalate (PEN)	Polyethylene Terephthalate (PET)	Liquid-Crystal Polymer (LCP)	Polydimethyl-Siloxane (PDMS)	Polyimide (PI)
Density (g/cm^3^)	1.39 [33]	1.41 [33]	1.38–1.95 [34]	0.95–1.08 [34]	1.06–1.45 [34]
Young’s modulus (MPa)	2000 [33]	1700 [33]	5000–20,000 [34]	0.36–0.87 [34]	1800–15,000 [34]
Poisson ratio	0.3–0.4 [2]	0.3–0.4 [2]	0.4 [35]	0.5 [2]	0.34 [2,36,37]
Glass transmission temperature (°C)	116–120 [33]	68–114 [33]	82–280 [34]	−125 [34]	290–430 [34]
Coefficient of thermal expansion (CTE) at 20 °C (ppm/K)	10–14 [2]	40–50 [2]	4–38 [34]	180–450 [34]	3–50 [34,36]
Moisture absorption (%)	0.3 [38]	0.4 [38]	0.02–0.04 [34]	0.1–1.3 [34]	2–4 [34]
Challenges	Lower thermal stability [26]	Lower thermal stability [26]	Lack of self-adhesion to metal [12,34]	High gas permeability [34,39]	High moisture-uptake, short of rigidity [34,40]

**Table 2:** Illustration of the strain based on defined combination of thicknesses and bending radii.

Substrate Thickness (µm)	Structure Thickness (µm)	Bending Radius (mm)	Strain (%)
25	0.2	1	1.26
25	2	5	0.27
25	5	10	0.15
50	0.5	1	2.53
50	5	5	0.55
50	10	10	0.30

## Data Availability

Not applicable.
